# Association of Generic Competition With Price Decreases in Physician-Administered Drugs and Estimated Price Decreases for Biosimilar Competition

**DOI:** 10.1001/jamanetworkopen.2021.33451

**Published:** 2021-11-15

**Authors:** Sean R. Dickson, Tyler Kent

**Affiliations:** 1 West Health Policy Center, Washington, DC; 2 West Health Institute, La Jolla, California

## Abstract

**Importance:**

Price decreases of biologic and biosimilar products in Medicare Part B have been minimal, even with biosimilar competition. Medicare reimburses clinicians for biologics and biosimilars differently than for brand-name and generic drugs, which has generated greater price reductions.

**Objective:**

To characterize the nature of price competition among brand-name and generic drugs under Medicare Part B and to estimate the cost savings to the program of subjecting biologic and biosimilar therapies to a similar price competition.

**Design, Setting, and Participants:**

This cohort study analyzed all brand-name drugs and their approved generic versions as well as biologics and biosimilars that were reimbursed under Medicare Part B from quarter 1 of 2005 to quarter 2 of 2021. Two separate data sets were created: brand-name and generic drugs as well as biologics and biosimilars data sets. Brand-name products with generic versions that were introduced before 2005 were excluded, and so were vaccines.

**Exposures:**

Number of generic and biosimilar competitors over time.

**Main Outcomes and Measures:**

Price change as a percentage of the brand-name drug or biologic price in the quarter before generic or biosimilar competition. Price change was modeled using a linear, fixed-effects time series regression, with the number of generic or biosimilar competitors as the main covariate. Time was expressed as the number of quarters since the first generic or biosimilar competitor entered the market. Savings were estimated by projecting the regression model of brand-name and generic drug competition to observed biologic and biosimilar competition and by applying the estimated price reduction to actual Medicare spending for those products from 2015 to 2019.

**Results:**

Of the 988 Healthcare Common Procedure Coding System codes identified, 50 (5.0%) met the inclusion criteria for the brand-name and generic drug data set and 28 (2.8%) met the criteria for the biologic and biosimilar data set. The first generic competitor was associated with reduced drug prices by 17.0%, the second competitor with a 39.5% decrease, the third competitor with a 52.5% decrease, and the fourth and more competitors with a 70.2% decrease (price decline was measured from brand-name drug price before the first generic competitor rather than from price established with fewer competitors). If biologics and biosimilars were subject to the same Medicare reimbursement framework as brand-name and generic drugs, Medicare spending on these products was estimated to have been 26.6% lower ($1.6 billion) from 2015 to 2019.

**Conclusions and Relevance:**

This study found minimal uptake of biosimilars and limited price reductions for biologics and biosimilars under the current Medicare Part B reimbursement policy. Adopting the bundled biosimilar reimbursement structure for biologic and biosimilar therapies may be associated with substantial savings and encourage greater biosimilar market entry.

## Key Points

**Question:**

Is generic drug competition associated with prices of physician-administered drugs, and what price changes could occur under increased biosimilar competition?

**Findings:**

In this cohort study of 50 brand-name drugs and generic versions as well as 28 biologics and biosimilars, generic competition was associated with reduced prices, achieving a nearly 53% price decrease after 3 generic competitors were approved. If biosimilar products were treated similar to generic products in the Medicare Part B program, spending on biologics with their approved biosimilars was estimated to have been nearly 27% lower from 2015 to 2019.

**Meaning:**

Findings from this study suggest that implementing the bundled biosimilar reimbursement model may be associated with substantially reduced Medicare spending and increased biosimilar market entry.

## Teaser

This cohort study examines the application of a combined reimbursement model to brand-name and generic drugs as well as biologic and biosimilar therapies in the Medicare Part B program.

## Introduction

Biologic therapies, which are complex combinations of sugars, proteins, and/or nucleic acids, account for most of Medicare Part B’s prescription drug spending and spending growth, reaching 80% of spending in 2017 and 92% of spending growth from 2006 to 2017.^1^ Despite these figures, biologics in the Medicare Part B program are not subject to direct price competition from biosimilar therapies. This situation differs from that for brand-name and generic drugs in the Medicare Part B program, wherein reimbursement is structured to encourage price competition.

Because of past policy choices, Medicare Part B reimbursement for biologics and biosimilars does not incorporate the price competition framework used for brand-name and generic drugs. Brand-name and generic drugs are reimbursed at 106% of the weighted average sales price (ASP)^2^ of the brand and all approved generic products, incorporating the lower generic drug prices into the reimbursement and encouraging clinicians to select the lowest cost option.^3^ Biologics and biosimilars, however, are each reimbursed at 106% of their own ASP, encouraging clinicians to select the highest cost option for the greatest reimbursement.^1^ Previously, under the Administration of former US president Barack Obama, biosimilars were reimbursed on the basis of a weighted ASP to promote competition among biosimilars (but not with biologics).^4^ This policy was revised by the Administration of then-president Donald Trump on the grounds that it had reduced the reimbursement for biosimilars, resulting in the current separate reimbursement for each biologic and biosimilar therapy (Table 1).^5^ Under this policy structure, however, biosimilar uptake remains low and prices remain high, differing from the experience in the US Department of Veterans Affairs and European health care systems.^6,7,8,9,10,11^ For example, in quarter 3 of 2018, Remicade (infliximab) still maintained 81% of the Medicare Part B market share of Remicade and 2 approved biosimilars, Inflectra (infliximab-dyyb) and Renflexis (infliximab-abda), even though the biosimilars were priced at a 17% to 23% discount off of the Remicade cost.^12^

**Table 1.** Biosimilar Reimbursement Policies Under the Obama and Trump Administrations vs Authors’ Policy Proposal and Example Reimbursement Structure^a^

	Obama Administration	Trump Administration	Authors’ proposal
Period	2009 to quarter 1 in 2018	Quarter 2 in 2018 to present	NA
Reimbursement policy	Innovator biologics separately reimbursed; biosimilars grouped into a single reimbursement code	Innovator biologics and biosimilars individually reimbursed under separate codes	Both innovator biologics and biosimilars reimbursed under a single code
Period	Quarter 1 in 2018	Quarter 2 in 2018	Proposed for quarter 2 2018
Example reimbursement coding structure			
HCPCS code: J1745	Remicade (infliximab); ASP: $85.81	Remicade (infliximab); ASP: $83.29	Infliximab biologics and biosimilars; ASP: $54.07 (estimated)
HCPCS code: Q5102	Infliximab biosimilars; ASP: $75.52	NA	NA
HCPCS code: Q5103	NA	Inflectra (infliximab-dyyb); ASP: $69.71	NA
HCPCS code: Q5104	NA	Renflexis (infliximab-abda); ASP: $70.38	NA

Abbreviations: ASP, average sales price; HCPCS, Healthcare Common Procedure Coding System; NA, not applicable.

^a^ ASPs are as reported in the Medicare quarterly ASP file and include the add-on percentage payment. The estimated ASP in the authors’ proposal was generated from a regression model of price changes for brand-name and generic drugs in Medicare Part B, with the regression parameters applied to the biologic and biosimilar data to estimate the price with bundled competition.

Given this background, 2 key gaps remain in the literature. First, to our knowledge, no study has yet characterized the association of combined reimbursement of Medicare Part B brand-name and generic drugs with the rate of price decline over time and by the number of competitors, although similar investigations have been performed outside of the Medicare Part B program.^13,14^ Second, in the biologic and biosimilar market, the rate of price decreases and savings associated with combined reimbursement, which we called *bundled biosimilar reimbursement*, has not been estimated. Therefore, in this study, we aimed to characterize the nature of price competition among brand-name and generic drugs under Medicare Part B and to estimate the cost savings to the program of subjecting biologic and biosimilar therapies to a similar price competition.

## Methods

To model the cost savings associated with bundled biosimilar reimbursement, we first characterized the rate of reimbursement change under the bundled biosimilar reimbursement framework for brand-name and generic drugs. We then applied these parameters to the biologic and biosimilar market. Per the decision guidance of the US Department of Health and Human Services, this cohort study was exempt from institutional review board approval and informed consent because it did not involve health care records and used only data that were publicly available. We followed the Strengthening the Reporting of Observational Studies in Epidemiology (STROBE) reporting guideline.

### Data Sources and Study Sample

This analysis used 5 data sources: the Medicare Part B ASP reimbursement files from quarter 1 (January-March) of 2005 to quarter 2 (April-June) of 2021,^2^ the associated ASP crosswalk files,^2^ the Medicare Part B drug spending dashboard from 2010 to 2019,^15^ and the US Food and Drug Administration Orange Book^16^ and Purple Book.^17^ Data were organized at the Healthcare Common Procedure Coding System (HCPCS) code level. Each HCPCS code uniquely identifies the amount that Medicare will reimburse for a given drug or biologic product in a calendar quarter.

The study sample was limited to drugs and biologics that were reimbursed under Medicare Part B from quarter 1 of 2005 to quarter 2 of 2021 that had a generic or biosimilar competitor enter during the period. Brand-name products with generic versions that were introduced before 2005 were excluded from the model. Vaccines were excluded from the sample. The eTable in the Supplement is a list of all included drugs.

The data were structured as panel data on a quarterly basis, with quarterly ASP reimbursement and the number of drug manufacturers included in the HCPCS code each quarter. Two separate data sets were created: 1 for brand-name and generic drugs and 1 for biologics and biosimilars.

For the brand-name and generic drugs (that were approved after 2005) data set, the ASP crosswalk files were used to identify all formulations that were reimbursed under each HCPCS code. The Orange Book was used to identify the discrete number of drug manufacturers that marketed formulations under each HCPCS code. The number of manufacturers was always considered to be 1 until an Abbreviated New Drug Application was approved. Because each brand-name drug receives its own HCPCS code, multiple brand-name drugs within a HCPCS code denote the transfer of the drug from 1 manufacturer to another. By coding these instances as 1 manufacturer, we avoided double counting of manufacturers.

For the biologic and biosimilar data set, the panel data were organized by the HCPCS code for the innovator biologic product. The Purple Book was used to identify the number of manufacturers of biosimilar formulations of the biologics during each quarter. The ASPs for the innovator biologics and each of the biosimilars were recorded for each quarter.

Both data sets included the number of annual beneficiaries, total spending, and total utilization from the Medicare Part B drug spending dashboard for 2010 to 2019.

### Statistical Analysis

Data were organized and prepared for analysis in SAS Studio, version 3.71 (SAS Institute Inc). All analyses were performed from May 1 to June 10, 2021, using Stata, version 16 (StataCorp LLC).

#### Regression Analysis

We analyzed the overall pattern of price changes in the Medicare Part B program for all drugs with generic competition from 2005 to 2021. We used a linear, fixed-effects time series regression model to estimate the rate of price change since the introduction of a generic competitor and the number of generic competitors that were present. The data were organized as a panel grouped by the brand-name drug, and time was measured as the number of quarters since the first quarter of generic competition. This method allowed the regression model to report the marginal association of a 1-quarter increase in time with price. An interaction term was included for the number of drug manufacturers by quarter. The dependent variable was the cumulative percentage price change in the bundled ASP (for brand-name and generic drugs) compared with the brand-name drug’s ASP in the quarter before generic competition, which is consistent with established practice.^18^ Average marginal effects were projected for each number of generic competitors.

To describe a typical price change pattern for the existing bundled reimbursement of brand-name and generic drugs, we created a data set using the median number of quarters for the period with 2, 3, 4, and 5 or more manufacturers in the data. The parameters from the generic competition price regression model were used to estimate price changes over time according to the number of generic competitors per quarter. These parameters were also used to estimate price changes in the biologic and biosimilar data set under the bundled biosimilar reimbursement model.

The output from the regression model for percentage price change in the brand and generic market was used to estimate the price change in the biosimilar competition data; this price change was, in turn, applied to estimate the savings under the bundled biosimilar reimbursement model. The estimated new Medicare price under the bundled biosimilar reimbursement approach was applied to both the innovator biologics and the biosimilars for the relevant quarter. Annual utilization from the Medicare Part B drug spending dashboard was evenly amortized over the 4 quarters to estimate savings under the policy.

#### Sensitivity Analyses

Regression analysis was performed for all drugs in the data set as well as on a limited set of drugs that were used by at least 5000 Medicare beneficiaries in at least 1 year for which utilization data were available (2010-2019); a list of drugs that meet this criterion is shown in the eTable in the Supplement. Regression analyses were also performed that excluded outlier quarters on the basis of the number of quarters of competition with a given number of generic drug manufacturers; outliers were defined as those quarters of data with competitors that were greater than 2 median absolute deviations (MADs) outside of the number of quarters with that number of competitors.^19^ This approach excluded quarters wherein, after a period of substantial competition, some generic drug manufacturers exited the market and prices began to increase because only 1 or 2 manufacturers were in the market. A 2-MAD threshold was chosen instead of the typical 3 because at 3 MADs, less than 2% of quarters were excluded; at 2 MADs, 7.3% of quarters were excluded. In the regression model that included outliers, average marginal effects were estimated both inclusive and exclusive of outliers. Because of the inclusion of an interaction term, outliers had limited implications for model coefficients for a typical period of drug competition but could affect the average marginal effect that was calculated across the entire data set.

## Results

Over the study period, 988 unique HCPCS codes were identified. Of these codes, 50 (5.0%) met the inclusion criteria for the brand-name and generic drug data set, and an additional 28 HCPCS codes (2.8%) for innovator biologics (7 codes) and their approved biosimilars (21 codes) during the period were included in the biologic and biosimilar data set. In the generic competition data, the median number of quarters was 5 with 1 generic competitor, 4 with 2 competitors, 3 with 3 competitors, and 13 with 4 or more competitors.

### Price Changes

Table 2 presents the results of the regression analyses of price change after the introduction of generic competition. After excluding the outliers among all drugs in the study, the presence of 1 generic competitor was associated with a 14.9% mean price reduction in the bundled ASP, 2 generic competitors were associated with a 32.7% mean price reduction, and 3 generic competitors were associated with a 52.0% mean price reduction. Additional competitors were associated with a mean total price decrease of 68.6%; all estimated reductions were from the price of the brand-name drug in the quarter before generic competition and not from the price with fewer generic competitors. The regression models that were inclusive of outliers showed similar associations for all numbers of competitors when the average marginal effects were calculated exclusive of outliers, although the average marginal effects that included outliers were associated with attenuated price effects for 1 or 2 generic competitors because of the inclusion of periods in which prices increased after competitors left the market. For example, in the model of all drugs used by 5000 or more beneficiaries, when outliers were excluded from the average marginal effects, 1 generic competitor was associated with a mean price decrease of 17.0%, 2 competitors with a 39.5% price decrease, 3 competitors with a 52.5% price decrease, and 4 or more competitors with a 70.2% price decrease. However, when outliers were included, 1 generic competitor was associated with a price increase of 11.1%; this association was attributed to outlier drugs whose price increased several years after some generic manufacturers left the market.

**Table 2.** Fixed-Effects Linear Panel Regression Estimating Change in Bundled Average Sales Price Over Time, by Number of Generic Competitors, 2005-2021

Model sample	Model fit parameters					Estimated mean price reduction by number of generic competitors (average marginal effects)				
	No. of drug groups	No. of observations	Mean No. of observations per group	*R* ^2^	*F* statistic	Estimation sample	No. of generic competitors, %			
							1	2	3	≥4
All drugs	50	1488	29.8	0.718	<.001	All drugs	−9.0	−27.1	−44.0	−66.2
						Excluding outliers^a^	−16.8	−33.1	−50.8	−66.3
All drugs, excluding outlier quarters^b^	50	1380	27.6	0.438	<.001	Excluding outliers	−14.9	−32.7	−52.0	−68.6
Drugs used by ≥5000 Medicare beneficiaries in any year of sample	24	620	25.8	0.969	<.001	All drugs	11.1	−22.9	−37.9	−71.1
						Excluding outliers^a^	−17.0	−39.5	−52.5	−70.2
Drugs used by ≥5000 Medicare beneficiaries in any year of sample, excluding outlier quarters^b^	24	580	24.2	0.695	<.001	Excluding outliers	−17.4	−40.0	−52.9	−70.7

^a^ Regression model was based on all drugs, but average marginal effects were estimated after excluding outlier quarters of data.

^b^ Outlier quarters were quarters wherein the observed number of generic manufacturers in that quarter exceeded 2 median absolute deviations from the median quarter with that number of generic manufacturers. These quarters generally represent scenarios wherein the number of generic manufacturers has decreased after some manufacturers have left the market.

The Figure shows the price changes over time for a median drug in the data set, wherein the number of quarters with each number of generic competitors represents the median observed in the full data set. This Figure is based on a projection from the regression model that was limited to drugs used by 5000 or more Medicare beneficiaries and included outliers, as this was the regression model with the greatest *R*^2^ (*R*^2^ = 0.969 vs *R*^2^ = 0.718 [the next greatest]) (Table 2).

**Figure.**
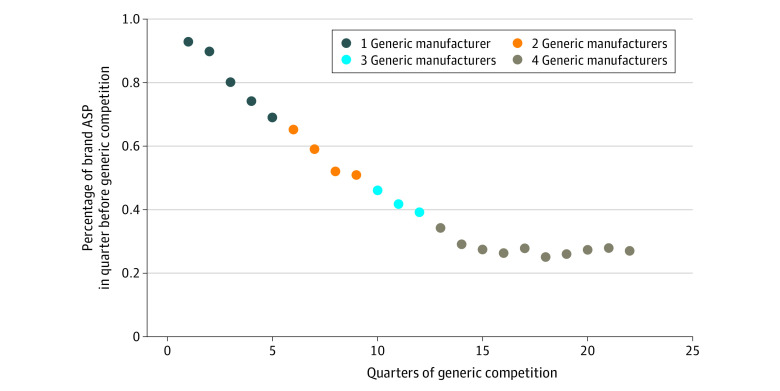
Estimated Percentage Change in Bundled Average Sales Price (ASP) by Quarter Since First Generic Competitor and Number of Generic Competitors, 2005-2021 The figure shows the projected fixed-effects linear panel regression model of the change in the bundled ASP for brand-name drug and generic drug competitors in the Medicare Part B program from 2005 to 2021. The number of quarters with each number of generic competitors represents the median number of quarters of competition with each number of generic competitors from the 50 unique chemical entities that had the first generic version approved from 2005 to 2021. The model was based on the 24 unique chemical entities that were used by 5000 or more Medicare beneficiaries in any year in which data were available (2010-2019); outliers were not excluded (Table 2).

### Savings Estimate

During the modeling period of 2015 to 2019 and for those years with reported Medicare spending on both biologics and their associated biosimilars, Medicare and its beneficiaries spent $6.5 billion on these 6 biologics with their biosimilar versions: Neupogen (filgrastim), Remicade (infliximab), Neulasta (pegfilgrastim), Avastin (bevacizumab), Herceptin (trastuzumab), and Epogen (epoetin alfa). Using the brand-name and generic drugs bundled reimbursement regression model that was limited to drugs used by 5000 or more beneficiaries and inclusive of outliers, we estimated the price change parameters of these 6 biologics and their biosimilars; all biologic and biosimilar therapies were used by 5000 or more beneficiaries. We estimated that the bundled biosimilar reimbursement model would have been associated with reduced spending on these therapies of $1.6 billion, or 26.6% (Table 3). Most of these savings were from decreases in spending on innovator biologics ($1.1 billion), but spending on biosimilars would have been half a billion lower as well. Estimated savings were greatest for Remicade (infliximab), at $3.6 billion (27.3%), for which 4 biosimilar versions were approved during the modeling period. A full list of included biologics is shown in Table 3.

**Table 3.** Estimated Medicare Part B Savings From the Bundled Biosimilar Reimbursement Model, 2015-2019

Biologics	Savings by year, $^a^					Total savings, $	Total spending for years with approved biosimilars, 2015-2019, $	Estimated savings, 2015-2019, %
	2015	2016	2017	2018	2019			
Neupogen (filgrastim)								
Total savings	2 371 451	24 381 448	26 535 802	15 987 418	24 947 564	94 223 683	394 782 560	23.9
Biologic savings	2 361 888	20 152 981	21 556 312	15 939 183	16 573 358	76 583 722	NA	NA
Biosimilar savings	9563	4 228 467	4 979 490	48 235	8 374 206	17 639 961	NA	NA
No. of biosimilars approved	1	1	1	1	2	NA	NA	NA
Remicade (infliximab)								
Total savings	NA	NA	246 211 952	500 842 400	231 704 224	978 758 576	3 585 824 512	27.3
Biologic savings	NA	NA	246 211 952	481 976 546	229 275 833	957 464 331	NA	NA
Biosimilar savings	NA	NA	NA	18 865 854	2 428 391	21 294 245	NA	NA
No. of biosimilars approved	NA	NA	1	3	4	NA	NA	NA
Neulasta (pegfilgrastim)								
Total savings	NA	NA	NA	2 527 827	500 637 600	503 165 427	1 748 490 496	28.8
Biologic savings	NA	NA	NA	2 077 786	25 474 592	27 552 378	NA	NA
Biosimilar savings	NA	NA	NA	450 041	475 163 008	475 613 049	NA	NA
No. of biosimilars approved	NA	NA	NA	1	2	NA	NA	NA
Epogen (epoetin alfa)								
Total savings	NA	NA	NA	850 276	3 714 835	4 565 111	337 709 536	1.4
Biologic savings	NA	NA	NA	850 651	3 184 624	4 035 275	NA	NA
Biosimilar savings	NA	NA	NA	−375	530 211	529 836	NA	NA
No. of biosimilars approved	NA	NA	NA	1	1	NA	NA	NA
Avastin (bevacizumab)								
Total savings	NA	NA	NA	NA	19 517 800	19 517 800	264 879 664	7.4
Biologic savings	NA	NA	NA	NA	20 052 544	20 052 544	NA	NA
Biosimilar savings	NA	NA	NA	NA	−534 744	−534 744	NA	NA
No. of biosimilars approved	NA	NA	NA	NA	1	NA	NA	NA
Herceptin (trastuzumab)								
Total savings	NA	NA	NA	NA	14 557 289	14 557 289	203 824 672	7.1
Biologic savings	NA	NA	NA	NA	15 008 166	15 008 166	NA	NA
Biosimilar savings	NA	NA	NA	NA	−450 877	−450 877	NA	NA
No. of biosimilars approved	NA	NA	NA	NA	1	NA	NA	NA
Overall								
Overall savings	2 371 451	24 381 448	272 747 754	520 207 921	795 079 312	1 614 787 886	6 066 807 104	26.6
Biologic savings	2 361 888	20 152 981	267 768 264	500 844 166	309 569 117	1 100 696 416	NA	NA
Biosimilar savings	9563	4 228 467	4 979 490	19 363 755	485 510 195	514 091 470	NA	NA

Abbreviation: NA, not applicable.

^a^ Unless otherwise indicated.

Although Medicare utilization data were available only through 2019, we projected the total decrease in the bundled ASP up to quarter 2 of 2021 (Table 4) and compared the estimated with the observed price changes in ASP for both the biologics and approved biosimilars. For 6 of the 7 biologics, the observed price in quarter 2 of 2021 exceeded the projected price under the bundled biosimilar reimbursement model; across all 7 biologics, the observed price was a mean of 47.5% greater than the projected price. Among the biosimilars, we calculated the arithmetic mean of the observed price of each approved biosimilar drug. Mean biosimilar prices exceeded the projected prices by a mean of 4.6%, although the biosimilars for Rituxan (rituximab) and Neupogen (filgrastim) were priced below the projections, at a savings of 31.7% and 7.8%, respectively.

**Table 4.** Comparison of Projected Decrease in Average Sales Prices as a Percentage of Biologic Price in Quarter Before Biosimilar Approval and Observed Average Sales Prices in Quarter 2 of 2021^a^

Biologic	No. of approved biosimilars	Projected decrease in bundled biologic and biosimilar ASP, %	Projected bundled biologic and biosimilar ASP, $	Observed biologic ASP, $	Difference in observed and projected biologic ASP in quarter 2 of 2021		Observed mean biosimilar ASP, $	Difference in observed and projected biosimilar ASP in quarter 2 of 2021	
					$	%		$	%
Neupogen (filgrastim)	2	54.9	0.45	0.95	0.50	111.4	0.41	–0.03	–7.8
Remicade (infliximab)	4	61.1	32.27	41.95	9.69	30.0	32.89	0.62	1.9
Neulasta (pegfilgrastim)	4	64.2	1691.82	2808.06	1116.24	66.0	2534.24	842.41	49.8
Avastin (bevacizumab)	2	40.9	48.00	72.51	24.51	51.1	55.12	7.11	14.8
Rituxan (rituximab)	3	34.8	61.77	89.14	27.37	44.3	42.19	–19.58	–31.7
Herceptin (trastuzumab)	5	34.4	70.20	93.68	23.49	33.5	71.08	0.88	1.3
Epogen (epoetin alfa)	1	31.8	0.89	0.85	–0.03	–3.7	0.92	0.03	3.6
Mean difference between observed and projected ASP	NA	NA	NA	NA	NA	47.5	NA	NA	4.6

Abbreviations: ASP, average sales price; NA, not applicable.

^a^ Under the authors’ proposed policy, biologics and their approved biosimilars would have a single ASP, weighted by use of each component. Because utilization data were not available for quarter 2 of 2021, this weighting with observed sales was not performed. The observed mean decrease for biosimilars was the arithmetic mean of all approved biosimilars and was not weighted by use.

Because price decreases are a function of both the number of competitors and time, drugs such as Remicade (infliximab), which has only 4 biosimilar competitors, have greater projected price declines (61.1%) compared with drugs with more approved biosimilars but less time in competition, such as Herceptin (trastuzumab), which has 5 approved biosimilars but only a 34.4% price reduction.

## Discussion

Findings from this study suggested that the current Medicare reimbursement policy for biologics and their approved biosimilars did not have the same magnitude of price competition as that observed for brand-name drugs and their approved generic versions. Under the current policy, Medicare spending on 6 biologics and their approved biosimilars was estimated to be $1.6 billion greater from 2015 to 2019 than the possible expenditure had Medicare implemented the bundled biosimilar reimbursement model.

The current reimbursement policy for biologics and biosimilars did not appear to have the level of product transition as that observed in the brand-name and generic drug market,^18^ and existing biosimilars were priced at a lower discount from the cost of innovator biologics than typically observed in pricing new generic entrants for physician-administered brand-name drugs. Findings from this study suggested that aligning the Medicare reimbursement structure for biologics and biosimilars with the existing reimbursement structure for brand-name and generic drugs was associated with the uptake of biosimilars and price competition. Other countries with a reimbursement policy, such as our proposed model, that creates a financial incentive for the uptake of biosimilars have seen greater switching and lower overall prices for biologics and biosimilars.^20^ The price-reduction estimates in the present study were somewhat greater than the findings from the nonphysician-administered market, which reported a 13% price reduction with 1 competitor (vs 17.0% in this study), 23% reduction with 2 competitors (vs 39.5% in this study), and 40% reduction with 3 competitors (52.5% in this study). A median of 64.0% price decrease was also found with 5 to 10 or more competitors (vs 70.2% for ≥4 competitors in this study).^13^ We hypothesized that the additional revenue spread achieved by clinicians when selecting a lower-priced product in the bundled reimbursement model may be associated with greater price decreases than seen in the pharmacy market.

The savings estimate under the bundled biosimilar reimbursement model was relatively low because the savings were historical rather than forward looking. As shown in Table 4, in the 18 months after the modeling period (quarter 1 in 2020 to quarter 2 in 2021), substantial additional price declines were estimated, reflecting the additional entry of new biosimilars and the maturation of competition within the market. A self-described back-of-the-envelope savings estimate of a related least costly alternative policy projected a savings of $1 billion to $7.5 billion in 2020, suggesting the magnitude of forward-looking savings.^21^ This potential for substantial greater future savings emphasizes the importance of rapidly implementing the bundled biosimilar reimbursement model to take full advantage of the price reductions that are associated with new biosimilar entrants. Moreover, rapid adoption would encourage additional biosimilar development, as biosimilars would be better able to compete on price under the bundled biosimilar reimbursement model compared with the current policy.

Adoption of the bundled biosimilar reimbursement model would also be associated with substantial cost savings for Medicare beneficiaries. Under the Medicare Part B program, beneficiaries are responsible for 20% of a drug or biologic cost.^22^ Of the $1.6 billion in savings estimated in the modeling period, approximately $1.3 billion would accrue to the Medicare Part B program, whereas the remaining $0.3 billion would offset beneficiary cost sharing. Although most beneficiaries do not pay these costs directly and instead finance their Medicare Part B cost-sharing obligations through Medigap insurance policies, the reductions in payments made by Medigap plans would eventually reach beneficiaries through lower premiums. Beneficiaries would also likely see lower Medicare Part B premiums.

Reimbursement policy alone will not solve all barriers to biosimilar entry into the market and uptake, and additional reforms that will encourage competition have been suggested.^23^ The pharmaceutical industry continues to argue that the current separate billing code system for biologics and biosimilars is necessary to adequately incentivize biosimilar development and marketing.^24^ However, given the relatively low uptake of biosimilars in the Medicare Part B program and the lack of incentives for drug manufacturers to compete on price, the current policy appears to be insufficient in encouraging appropriate competition and price reduction.

### Limitations

This study has several limitations. First, biologics and biosimilars may not compete on price in the same way that brand-name and generic drugs may, which has implications for the magnitude of the savings estimates. In a related least costly alternative policy for prostate cancer treatments, however, competing brand-name drug manufacturers took sharp price decreases to maintain market share,^25^ suggesting the presence of an incentive for price competition under the bundled biosimilar reimbursement model. Second, current biosimilars may not be completely interchangeable with existing biologics, which may change clinicians’ ability to select a lower-cost product for a given patient. This concern is generally limited to individual cases, however, and is unlikely to affect the magnitude of the savings estimates. Third, the regression model we used transformed all data to count the time from the first quarter of generic or biosimilar competition, which may overlook market impacts during the actual date of transition. However, the use of a fixed-effects regression model ameliorates these concerns. Fourth, because the regression analysis was designed to estimate the mean change in price over time with generic competition in the Medicare Part B program and to not generate a parameter estimate for the marginal effect of additional competition, we did not account for serial association. Given that the data represented the entirety of the transition from brand-name to generic products in the Medicare Part B program and not a sample, we believe that the general concern about serial association (that it differs in the sample from the broader population) is not applicable.

## Conclusions

In this cohort study, the current Medicare Part B reimbursement policy for biologic and biosimilar therapies appeared to be associated with minimal uptake of biosimilars and limited price reductions for biologic and biosimilar products. We estimated that the bundled biosimilar reimbursement model would have been associated with reduced spending (by nearly 27% or $1.6 billion) on 6 biologics from 2015 to 2019. Adopting this model could produce substantial savings and encourage additional biosimilar market entry.

## Notes

Supplement. **eTable.** Physician-Administered Drugs Included in Regression Model of Price Change Following Generic Transition, 2005-2021

## References

[1] Office of the Assistant Secretary for Planning and Evaluation, US Department of Health and Human Services. Medicare Part B drugs: trends in spending and utilization, 2006-2017. Accessed June 2, 2021. https://aspe.hhs.gov/system/files/pdf/264416/Part-B-Drugs-Trends-Issue-Brief.pdf 39146449

[2] Centers for Medicare & Medicaid Services. 2021 ASP drug pricing files. Accessed May 10, 2021. https://www.cms.gov/medicare/medicare-part-b-drug-average-sales-price/2021-asp-drug-pricing-files

[3] US Government Accountability Office. Medicare Part B: CMS should take additional steps to verify accuracy of data used to set payment rates for drugs. GAO-16-594. July 2016. Accessed June 2, 2021. https://www.gao.gov/assets/gao-16-594.pdf

[4] Centers for Medicare & Medicaid Services. Payment policies under the physician fee schedule and other revisions to Part B for CY 2011, CMS-1503-FC. *Fed Regist*. 2010;75:73393-73394. Codified at 42 CFR §414.902-904. Accessed May 26, 2021. https://www.govinfo.gov/content/pkg/FR-2015-11-16/pdf/2015-28005.pdf

[5] Centers for Medicare & Medicaid Services. Revisions to payment policies under the physician fee schedule and other revisions to Part B for CY 2018; Medicare shared savings program requirements; and Medicare diabetes prevention program, CMS-1676-F. Fed Regist. 2017;82:53182-531187. 29231695

[6] Chen AJ, Gascue L, Ribero R, Van Nuys K. Uptake of infliximab biosimilars among the Medicare population. JAMA Intern Med. 2020;180(9):1255-1256. doi:10.1001/jamainternmed.2020.3188 32702080 PMC7372498

[7] Kozlowski S, Birger N, Brereton S, . Uptake of the biologic filgrastim and its biosimilar product among the Medicare population. JAMA. 2018;320(9):929-931. doi:10.1001/jama.2018.9014 30193265 PMC6142991

[8] Dean EB, Johnson P, Bond AM. Physician, practice, and patient characteristics associated with biosimilar use in Medicare recipients. JAMA Netw Open. 2021;4(1):e2034776. doi:10.1001/jamanetworkopen.2020.34776 33502485 PMC7841457

[9] Socal MP, Anderson KE, Sen A, Bai G, Anderson GF. Biosimilar uptake in Medicare Part B varied across hospital outpatient departments and physician practices: the case of filgrastim. Value Health. 2020;23(4):481-486. doi:10.1016/j.jval.2019.12.007 32327165 PMC8875277

[10] Baker JF, Leonard CE, Lo Re V III, Weisman MH, George MD, Kay J. Biosimilar uptake in academic and Veterans Health Administration settings: influence of institutional incentives. Arthritis Rheumatol. 2020;72(7):1067-1071. doi:10.1002/art.41277 32253823 PMC7329608

[11] Kim Y, Kwon HY, Godman B, Moorkens E, Simoens S, Bae S. Uptake of biosimilar infliximab in the UK, France, Japan, and Korea: budget savings or market expansion across countries? Front Pharmacol. 2020;11:970. doi:10.3389/fphar.2020.00970 32733238 PMC7363801

[12] Medicare Payment Advisory Commission. Report to Congress: Medicare and the health care delivery system. Medicare payment strategies to improve price competitions and value for Part B drugs. June 2019. Accessed June 2, 2021. http://www.medpac.gov/docs/default-source/reports/jun19_ch3_medpac_reporttocongress_sec.pdf?sfvrsn=0

[13] Dave CV, Hartzema A, Kesselheim AS. Prices of generic drugs associated with numbers of manufacturers. N Engl J Med. 2017;377(26):2597-2598. doi:10.1056/NEJMc1711899 29281576

[14] Conrad R, Lutter R. Generic competition and drug prices: new evidence linking greater generic competition and lower generic drug prices. December 2019. Accessed October 7, 2021. https://www.fda.gov/about-fda/center-drug-evaluation-and-research-cder/generic-competition-and-drug-prices

[15] Centers for Medicare & Medicaid Services. Medicare Part B drug spending dashboard. Accessed May 10, 2021. https://www.cms.gov/Research-Statistics-Data-and-Systems/Statistics-Trends-and-Reports/Information-on-Prescription-Drugs/MedicarePartB

[16] US Food and Drug Administration. Orange Book: approved drug products with therapeutic equivalence evaluations. Accessed May 10, 2021. https://www.accessdata.fda.gov/scripts/cder/ob/index.cfm

[17] US Food and Drug Administration. Purple Book: lists of licensed biological products with reference product exclusivity and biosimilarity or interchangeability evaluations. Accessed May 10, 2021. https://www.fda.gov/drugs/therapeutic-biologics-applications-bla/purple-book-lists-licensed-biological-products-reference-product-exclusivity-and-biosimilarity-or

[18] Stern AD, Chen JL, Ouellet M, . Biosimilars and follow-on products in the United States: adoption, prices, and users. Health Aff (Millwood). 2021;40(6):989-999. doi:10.1377/hlthaff.2020.02239 34097520

[19] Leys C, Ley C, Klein O, Bernard P, Licata L. Detecting outliers: do not use standard deviation around the mean, use absolute deviation around the median. J Exp Soc Psychol. 2013;49(4):764-766. doi:10.1016/j.jesp.2013.03.013

[20] Robinson JC, Jarrion Q. Competition from biosimilars drives price reductions for biologics in the French single-payer health system. Health Aff (Millwood). 2021;40(8):1190-1197. doi:10.1377/hlthaff.2021.00070 34339240

[21] Morton FS. Paying for Biologic PADS in Medicare Part B. onepercentsteps.com. Accessed June 2, 2021. https://onepercentsteps.com/policy-briefs/paying-for-biologic-pads-in-medicare-part-b/

[22] US Government Accountability Office. Medicare Part B: expenditures for new drugs concentrated among a few drugs and most were costly for beneficiaries. GAO-16-12. October 2015. Accessed June 2, 2021. https://www.gao.gov/assets/gao-16-12.pdf

[23] Van de Wiele VL, Kesselheim AS, Sarpatwari A. Barriers to US biosimilar market growth: lessons from biosimilar patent litigation. Health Aff (Millwood). 2021;40(8):1198-1205. doi:10.1377/hlthaff.2020.02484 34339242

[24] Hagen T. PhRMA: federal biosimilar payment policies are having a positive effect. March 4, 2020. Accessed June 2, 2021. https://www.centerforbiosimilars.com/view/phrma-federal-biosimilar-payment-policies-are-having-a-positive-effect

[25] Committee for a Responsible Federal Budget. Injecting price competition into Medicare Part B drugs. Published July 26, 2021. Accessed July 26, 2021. https://www.crfb.org/papers/injecting-price-competition-medicare-part-b-drugs

