# Supplementary material for: Association of Generic Competition With Price Decreases in Physician-Administered Drugs and Estimated Price Decreases for Biosimilar Competition
Source: JAMA Netw Open. 2021 Nov 15;4(11):e2133451. doi: 10.1001/jamanetworkopen.2021.33451 (PMC8593762; doi:10.1001/jamanetworkopen.2021.33451)
Supplement: Supplement. — eTable. Physician-Administered Drugs Included in Regression Model of Price Change Following Generic Transition, 2005-2021 [file jamanetwopen-e2133451-s001.pdf]

Supplementary Online Content

Dickson SR, Kent T. Association of generic competition with price decreases in physician-administered drugs and estimated price decreases for biosimilar competition. *JAMA Netw Open*. 2021;4(11):e2133451. doi:10.1001/jamanetworkopen.2021.33451

eTable. Physician-Administered Drugs Included in Regression Model of Price Change Following Generic Transition, 2005-2021

This supplementary material has been provided by the authors to give readers additional information about their work.

© 2021 Dickson SR et al. *JAMA Network Open.*

eTable. Physician-Administered Drugs Included in Regression Model of Price Change Following Generic Transition, 2005-2021

HCPCS Code	Brand Drug Name	Generic Drug Name	Met the 5,000 Beneficiary Threshold
A9575	Dotarem	Gadoterate Meglumine	Yes
J0583	Angiomax	Bivalirudin	No
J0637	Cancidas	Caspofungin Acetate	No
J0641	Fusilev	Levoleucovorin Calcium	Yes
J0706	Cafcit	Caffeine Citrate	No
J0740	Vistide	Cidofovir	No
J0834	Cortrosyn	Cosyntropin	No
J0878	Cubicin	Daptomycin	Yes
J0894	Dacogen	Decitabine	No
J1120	Diamox	Acetazolamide Sodium	No
J1190	Zinecard	Dexrazoxane	No
J1325	Flolan	Epoprostenol	No
J1335	Invanz	Ertapenem	Yes
J1451	Antizol	Fomepizole	No
J1453	Emend	Fosaprepitant	Yes
J1455	Foscavir	Foscarnet Sodium	No
J1570	Carimune	Immune Globulin	No
J1570	Cytovene	Ganciclovir	No
J1652	Arixtra	Fondaparinux Sodium	No
J1740	Boniva	Ibandronate sodium	Yes
J1742	Corvert	Ibutilide Fumarate	No
J1810	Innovar	Droperidol/Fentanyl Citrate	No
J1953	Keppra	Levetiracetam	No
J1956	Levaquin	Levofloxacin	No
J2020	Zyvox	Linezolid	No
J2185	Merrem	Meropenem	No
J2210	Methergine	Methylergonovine Maleate	No
J2354	Sandostatin (non-depot)	Octreotide Acetate	No
J2501	Zemplar	Paricalcitol	No
J2543	Zosyn	Ampicillin Sodium	No
J2780	Zantac	Ranitidine Hydrochloride	Yes
J2795	Naropin	Ropivacaine Hydrochloride	Yes
J2916	Ferrlecit	Sodium Ferric Gluconate	Yes
J3243	Tygacil	Tigecycline	No

© 2021 Dickson SR et al. *JAMA Network Open.*

J3465	Vfend	Voriconazole	No
J7507	Prograf	Tacrolimus	Yes
J7517	Cellcept	Mycophenolate Mofetil	Yes
J7518	Myfortic	Mycophenolic Acid	Yes
J7520	Sirolimus	Rapamune	Yes
J7612	Xopenex	Levalbuterol	No
J9025	Vidaza	Azacitidine	Yes
J9171	Taxotere	Docetaxel	Yes
J9178	Ellence	Epirubicin	No
J9201	Gemzar	Gemcitabine Hydrochloride	Yes
J9206	Camptosar	Irinotecan Hydrochloride	Yes
J9263	Eloxatin	Oxaliplatin	Yes
J9330	Torisel	Temsirolimus	No
J9351	Hycamtin	Topotecan Hydrochloride	No
J9357	Valstar	Valrubicin	No
J9395	Faslodex	Fulvestrant	Yes
Q2009	Cerebyx	Fosphenytoin Sodium	No

Note: Regression modeling was performed on all drugs as well as limited to drugs that were used by at least 5,000 Medicare beneficiaries during any single year for which utilization data were available (2010-2019).

© 2021 Dickson SR et al. *JAMA Network Open.*
